# Spring Cleaning in Small Institutions

**Published:** 1912-05-11

**Authors:** 


### INSTITUTIONAL HOUSEKEEPING.

### Spring Cleaning in Small Institutions.

Spring cleaning is always an anxious time for the matron, but careful preparation and forethought will enable her to disturb the ordinary routine as little as possible and thus minimise its discomforts.

The late spring or early summer is the best time to choose, as the days are longer and fewer fires are necessary—institutions are often free from the winter pressure on beds at this season—while winter blankets, etc., may be put away, and in the sitting-rooms the lighter covers and curtains may replace the winter ones.

To prepare for spring cleaning, all redecoration and renovation must be decided on, and arrangements made with workpeople. Extra help must be engaged: charwomen for cleaning furniture, floors, and paint, and a man for washing the walls. Domestics can sometimes be spared from the other parts of the building, and in certain places it may be possible to manage without outside help, but if it is likely to cause discontent among the domestic staff it is much better to engage one or two helpers. Arrangements must be made for beating and cleaning carpets, if there are any; all lockers, cupboards, fittings to doors and windows, etc., must be examined and mended. The chimney-sweep must be engaged, and whatever the rule about sweeping, on this occasion he should visit every chimney in the house. All cleaning materials and utensils must be reviewed, and the careful arrangement of work for each cleaner planned out. Inventories of stores, linen and china should be carefully examined, and the stock made up, each person in charge of the different departments of the household to be responsible under the matron's supervision. All worn-out and old bed-linen, etc., must be either disposed of or made use of for another purpose. Winter curtains must be taken down, and shaken well; if woollen they should be rubbed with hot bran to take out the stains, and then hung out before packing, in plenty of paper to prevent creases, with some moth-preventive—camphor, Keating's powder, carbon, naphthaline, strips of calico in turpentine or pepper, or Russia leather shavings. Blankets not required must be washed and stored like curtains. When all preparations are ready the actual cleaning may be begun. The order for cleaning should be from top to bottom, the farthest room from the staircase first. The patients must if possible be moved into another ward, and too many rooms should not be attempted at the same time. Meals for the workpeople should be plentiful, nourishing, and served punctually. All the furniture should be cleaned with vinegar and water or with water containing a weak disinfectant that will not harm the varnish, such as Izal or Cyllin, one tablespoonful to one gallon of water, and the lockers must be scrubbed inside, using hot water, soap, and a disinfectant. Small furniture must be moved out of the room, and the large furniture placed in the centre of the room, covered with dust sheets. All blinds should be taken down and cleaned; for Venetians the tapes are unfastened and the laths unstrung; the tapes and laths are washed in warm, soapy water, pinning the tapes out to dry to prevent shrinkage; holland blinds are washed in bran water, starched and ironed, or sent to a cleaner's. The walls and ceilings must be swept down, then washed with a disinfectant and water, or sprayed if not washable. All the beds should be taken to pieces, and all parts cleaned; the mattresses thoroughly beaten, and hung out, if possible, in the air; the iron parts cleaned with a rag dipped in turpentine or paraffin. Unvarnished paint must be washed with warm water in which Lux or shredded, dissolved soap has been placed, with a little disinfectant. Varnished paint must be sponged with borax and water—two tablespoonfuls dissolved in a little boiling water added to one gallon of cold water—and well rinsed. The furniture must next be polished, the floor cleaned thoroughly, and the windows well washed. The furniture is then replaced and the room left for a few hours thoroughly to dry before it is occupied. Sitting-rooms are cleaned in much the same manner, only that all ornaments and picture-glasses must be washed, and the frames polished. Gilt frames may be brightened with a mixture made by boiling three onions in a pint of water till they are soft and adding one tablespoonful of sulphur.

### Housekeeping Queries.

#### Plague of Flies.

Sister T.—*Our larder is infested with tiny flies. They seem to get in everywhere. How can we get rid of them?*

You give so little detail, it is difficult to judge the cause. We should suggest: (1) Cut away any ivy or creeper round the neighbourhood of the window, and, if possible, any overhanging trees, especially larch; (2) ascertain if there is any decaying rubbish in the vicinity of the larder; (3) fill all holes and cracks in woodwork, walls, floors, etc., with putty or cement; (4) have ceiling and walls scraped, then whitewashed, and floors, shelves, window, etc., washed with disinfectant, not forgetting the meshes in any ventilator in wall, door, or window. After this treatment flies should be a thing of the past.

#### Lemon Rinds.

Thrifty One.—*Large quantities of lemonade are made daily for our nurses; we use fresh lemons, not essence. Could you suggest any way of utilising the rinds after squeezing out the juice?*

The rinds should be used for the lemonade if you desire to be economical. If an infusion is made it is also infinitely nicer than merely squeezing the juice into water. The rinds should be chipped off thinly, so that the pieces are yellow on both sides. Then put them into a jug, add either the lemons, cut in thin slices after removing the pith and pips, or, if in a hurry, the strained juice. Pour in a quart of boiling water for four lemons and add about an ounce of sugar. Cover tightly till co[illegible] then strain off and use.

#### To Our Readers.

In this column questions are dealt with relating to cookery and household recipes, the duties of the domestic staff, estimates of quantities required for given numbers, and the cost of diet for patients and staff. Arrangements have been made to deal with samples submitted for examination and report on them.

